# The Complication Consult: A Plastic Surgery Perspective on Etiquette, Ownership, and Collegiality After Surgical Complications

**DOI:** 10.7759/cureus.113278

**Published:** 2026-07-24

**Authors:** Monali Mahedia, Andrew M Klapper, Anthony N Dardano, Barite Gutama, Alay Shah

**Affiliations:** 1 Plastic Surgery, Larkin Community Hospital, Miami, USA; 2 Plastic and Reconstructive Surgery, Florida Atlantic University Charles E. Schmidt College of Medicine, Boca Raton, USA; 3 Plastic Surgery, Mayo Clinic, Rochester, USA; 4 Plastic Surgery, New York University (NYU) Langone Health, New York, USA

**Keywords:** adverse event disclosure, consult etiquette, handoff communication, interprofessional communication, orthoplastic surgery, patient safety, plastic surgery, professionalism, surgical complications

## Abstract

Plastic surgeons are frequently asked to assist when postoperative complications cross service lines and create wound breakdown, soft-tissue necrosis, exposed hardware, infection, dead space, or reconstructive uncertainty. These consultations are often necessary, but their conduct can either preserve trust and collaboration or create a perceived transfer of blame and ownership. This editorial proposes a five-part etiquette framework for complication consultation: candor in naming the problem; courtesy through early, direct, attending-level communication; continuity through ongoing involvement of the index surgeon; completeness of clinical information and planning; and co-ownership of the patient’s care. The framework also emphasizes explicit consult questions, objective documentation, transparent patient communication, preservation of reconstructive options, and clear discharge responsibilities. Although presented from a plastic surgery perspective, these principles apply across surgical specialties. A complication consult should be understood as an escalation of care, not abandonment or transfer of inconvenience.

## Editorial

Why etiquette matters after a surgical complication

Surgeons are trained to manage technical problems, but the interpersonal culture that follows a complication is often less explicitly taught. When a wound breaks down, tissue becomes nonviable, hardware becomes exposed, infection threatens an implant, or closure is no longer durable, the clinical problem may appropriately require a plastic surgeon. The consultation itself is not the issue; rather, the issue is whether the consultation is conducted as a collegial escalation of care or as an unspoken transfer of worry, blame, inconvenience, and patient relationship.

The American College of Surgeons has articulated that the surgeon or surgical group remains responsible for postoperative care, including management of postoperative complications, and that even when aspects of care are delegated to others, the surgeon or group must remain involved in the care [1]. The language of this responsibility is important because complications often arise at the exact moment when patients most need continuity. The AMA Journal of Ethics has similarly discussed "turfing" as the offloading of responsibilities to others, noting its potential to harm patients and undermine collaboration [2]. From a plastic surgery perspective, the complication consult is therefore not merely a request for wound closure. It is a test of whether the surgical profession can behave as one team when the outcome is uncertain or disappointing. In practical terms, that test is whether the index surgeon names the complication, seeks appropriate expertise early, remains visible to the patient, and participates in the resulting plan rather than using consultation to distance the primary team from an adverse outcome.

The consult is not the transfer

Interhospital transfer has its own procedural, ethical, and regulatory requirements. This editorial focuses instead on the etiquette that occurs before, after, or apart from transfer: the bedside consult, the operating room request, the postoperative complication call, the shared clinic visit, the difficult family conversation, the handoff to the covering team, the discharge plan, and the follow-up after salvage. These moments are smaller than a formal transfer, but collectively they determine whether the patient experiences coordinated care or interservice fragmentation.

Name the complication honestly

The first etiquette obligation is candor. A vague consult for "wound evaluation" is inadequate when the actual concern is exposed plate, threatened flap, purulence, full-thickness necrosis, dehiscence under tension, dead space, vascular compromise, or the possible need for amputation. Euphemism protects the sender from discomfort but deprives the consultant of clinical context. The consult question should name the problem and the desired decision. For example, "Can you evaluate whether this exposed tibial hardware requires flap coverage after repeat debridement?" is better professional communication than "Plastics to assess wound." When limb salvage is uncertain, the consult should state that question directly. A severely injured or infected limb may be technically salvageable yet unlikely to provide useful function, while attempted salvage may impose unacceptable physiologic burden or repeated operations. Except in an immediate life-threatening emergency, a decision for delayed amputation should be based on multidisciplinary attending-level assessment, commonly involving orthopedic and plastic surgery and, when relevant, vascular surgery and rehabilitation, together with the patient and family. The purpose of each consultation should be explicit: to assess tissue viability, reconstructive feasibility, expected function, operative burden, rehabilitation potential, and whether salvage or amputation best serves the patient’s goals.

This principle applies equally in documentation and speech. Objective language should replace blame language. A note that describes exposed tendon, necrotic skin, persistent drainage, prior debridements, culture results, vascular findings, and current antibiotics helps the patient. A note that describes another service as having "failed" or "refused" care rarely improves care. Candor is not hostility. It is accurate naming of the clinical problem without theatrics.

Call early and at the right level

Many reconstructive failures begin before the formal consult is placed. A high-risk incision is closed under tension, a wound is repeatedly observed despite progressive skin loss, negative pressure therapy becomes a destination rather than a bridge, or debridements continue until local options have been exhausted. Early consultation is not a sign of weakness by the index surgeon; it is a form of planning. Institutions should consider explicit triggers for early plastic surgery consultation within complex-wound pathways. Such triggers may include exposed bone, tendon, nerve, vessel, or hardware; progressive tissue necrosis; threatened closure; substantial dead space; vascular compromise; recurrent dehiscence; or an anticipated need for graft, flap, or staged reconstruction. This is not intended to mandate plastic surgery involvement for every wound but to prevent delayed referral when reconstructive expertise could preserve options or reduce repeated ineffective interventions. A serious complication deserves attending-to-attending communication, particularly when operative timing, implant retention, flap coverage, limb salvage, or patient transfer is being considered.

Attending-level communication should not be viewed as bypassing residents or advanced practice clinicians. Trainees should participate and learn from these conversations, but the responsibility for a high-stakes consult should not be delegated entirely to the most junior person in the chain. The sending surgeon should be able to state the index procedure, operative date, timeline, prior attempts at management, current physiologic status, culture and imaging data, implants or hardware present, vascular status, anticoagulation, and the specific reason the plastic surgeon is being asked to help.

Ask an explicit question

A consult is a professional conversation, not an order. The most useful consults begin with a question. Does the wound need operative debridement? Is durable closure possible? Is flap coverage needed? Should hardware be retained, removed, exchanged, or temporized? Is local tissue usable? Is the patient a candidate for microsurgical reconstruction? Is an amputation discussion necessary? Should the case be done jointly? When the question is explicit, the consultant can answer it, document it, and explain it to the patient.

Continue ownership after asking for help

The phrase "plastics are taking over" is often inaccurate and potentially harmful. Plastic surgery may assume responsibility for soft-tissue reconstruction, flap monitoring, drains, grafts, and wound strategy, but the index surgeon often remains responsible for the bone, joint, implant, oncologic margin, vascular inflow, bowel leak, spine stability, trauma plan, or disease process that generated the reconstructive need. A more accurate message is "We are adding plastic surgery because soft-tissue reconstruction is essential, and I will continue to be involved in the overall surgical plan."

The same courtesy applies in the opposite direction. Plastic surgeons receiving a complication consult should not begin with public judgment. A complicated wound is rarely improved by humiliating the referring surgeon in the patient’s room, the operating room, the chart, or the hallway. The consultant should ask direct questions, clarify what is known, identify missing data, and state what must occur before reconstruction can proceed. Collegiality does not require silence about unsafe care. It requires that critique be delivered in the setting and language most likely to improve the patient situation.

Treat handoff as a safety intervention

Complication care commonly crosses shifts, teams, hospitals, and specialties. The Joint Commission has highlighted that handoff problems often arise from mismatched expectations between the sender and receiver of information, particularly when the receiver does not get what is needed for continuing care [3]. For a surgical complication, a handoff should not stop at "plastics following." It should include the operative timeline, current wound status, pending cultures, antibiotic plan, anticoagulation, activity restrictions, drain and dressing instructions, flap or graft monitoring needs, next operative contingency, and who will update the family if the plan changes.

The handoff should also identify the boundary of each service. The plastic surgeon may own flap checks, dressing changes, and drain removal; the orthopedic surgeon may own weight-bearing status and hardware decisions; infectious disease may own antibiotic duration; medicine may own optimization; and case management may own home health feasibility. Multidisciplinary care fails when every service assumes another service has explained the plan.

Communicate with the patient before colleagues lose the narrative

Patients know when teams are avoiding each other. They also know when a complication is being renamed in softer language. After an adverse event or unexpected outcome, communication should be timely, transparent, and compassionate. The Agency for Healthcare Research and Quality CANDOR process describes a structured approach for health care institutions and practitioners to respond in a timely, thorough, and just way when unexpected patient harm occurs [4]. In surgical practice, that principle means the patient should hear why plastic surgery is being involved, what is known, what remains uncertain, and what the next step is.

A respectful patient-facing script might be: "You have developed a serious wound and soft-tissue problem after surgery. I do not want to minimize it. I have asked a plastic surgeon to join your care because durable coverage and reconstruction are essential parts of treating this. I will continue to be involved, and we will make the plan together." This language avoids three common errors: pretending the problem is minor, implying that the index surgeon is disappearing, and promising that plastic surgery can simply "fix it."

Respect reconstructive planning before the operating room

Plastic surgery should not be treated as an intraoperative surprise for a predictable defect. When soft-tissue risk is foreseeable, consultation should occur before incision placement, definitive fixation, implant placement, tumor resection, radiated-field closure, or repeated debridement. Incisions, fasciotomy extensions, perforator territories, prior scars, vascular pedicles, exposed structures, and planned hardware all influence reconstructive options. A technically successful index operation can become a reconstructive failure if closure was never part of the plan.

The orthoplastic model offers a useful example. Open fracture standards emphasize consultant-level combined planning by orthopedic and plastic surgeons for fixation and coverage, timely definitive soft-tissue coverage, and avoidance of definitive internal stabilization unless durable soft-tissue cover can follow [5]. Although these standards address open fractures, the principle is broader: when bone, implant, wound, and coverage decisions are inseparable, the services should plan concurrently rather than sequentially.

Do not ask for coverage over an uncontrolled problem

One of the most common etiquette failures is asking plastic surgery to close a wound before the wound is ready. Durable reconstruction cannot substitute for source control, viable tissue, stable skeletal or implant strategy, adequate vascular inflow, and a realistic postoperative plan. A flap can fill dead space and bring vascularized tissue, but it cannot by itself solve unchecked purulence, unaddressed ischemia, unstable hardware, ongoing contamination, or a patient who cannot comply with postoperative protection. The courteous request is not "Can you close this today?" but rather "What has to be true before definitive closure is safe?"

This distinction protects patients and consultants. It also protects the index surgeon from the false reassurance that closure equals cure. In many cases, the best plastic surgery recommendation is not immediate reconstruction but staged debridement, additional imaging, vascular evaluation, culture-directed therapy, nutritional optimization, smoking cessation, pressure offloading, or joint decision-making about implant salvage versus removal.

Document facts, not resentment

The medical record is not the place for interservice sarcasm. Documentation should state the consult question, objective findings, patient status, communication between attending surgeons, shared decisions, contingency plans, and the parts of care owned by each team. A useful note describes what was seen, what was done, what is recommended, and who was informed. It does not need to encode blame.

This does not mean unsafe patterns should be ignored. If delayed consultation, missing records, incomplete debridement, absent imaging, or inappropriate discharge planning creates risk, the concern should be documented accurately and escalated through the appropriate quality, safety, or morbidity and mortality pathway. Professional restraint in the chart should not become professional silence about preventable harm.

Clarify discharge and follow-up ownership

Many complication consults fail after the operation, not during it. The patient leaves the hospital with unclear instructions about drains, dressings, splints, antibiotics, anticoagulation, elevation, dangling, weight-bearing, range of motion, showering, negative pressure therapy, flap monitoring, home nursing, and clinic follow-up. A discharge plan should specify which service handles which problem and whom the patient calls after hours. No patient should be asked to mediate between clinics to determine whether a concern belongs to the surgeon who created the wound, the plastic surgeon who reconstructed it, or the physician managing infection.

The follow-up courtesy extends to colleagues. After major salvage, the plastic surgeon should communicate what was found and what was done. The index surgeon should remain reachable and should participate in ongoing decisions about hardware, oncologic surveillance, rehabilitation, or additional procedures. Closing the loop is not merely polite; it prevents downstream misunderstanding.

Teach trainees the etiquette of complications

Residents and fellows learn not only from what attendings say but also from how attendings behave when a case becomes difficult. They notice whether the surgeon calls early or late, speaks directly or hides behind the chart, describes complications honestly or euphemistically, blames another service or asks for help, and updates the patient or avoids the room. If trainees are taught that "getting plastics on board" is equivalent to solving a problem, they will reproduce that culture. If they are taught that a consult creates shared responsibility, explicit questions, and follow-through, they will carry that culture forward.

Trainees should also be protected from becoming messengers for attending-level conflict. A junior resident should not be placed in the position of defending an incomplete consult, explaining a complication that the attending has not discussed with the patient, or negotiating a transfer of ownership between services. The attending surgeon who owns the decision should own the conversation.

The receiving plastic surgeon also has etiquette duties

Plastic surgeons have a corresponding obligation to receive consults professionally. The consultant should not assume negligence from an incomplete story, should not belittle another service in front of the patient, and should not overpromise salvage. The plastic surgeon should explain uncertainty clearly: reconstruction may be staged, infection may prevent immediate closure, tissue viability may declare itself over time, vascular workup may be necessary, and amputation or implant removal may become the safer option.

The consultant should also answer the question that was asked or explain why the question is incomplete. If the problem is not primarily reconstructive, the plastic surgeon should say so and identify what service or diagnostic step is needed. Courtesy does not require accepting inappropriate ownership. It requires helping define the correct ownership.

A practical language standard

The sending surgeon can set the tone with one sentence: "I am calling about my patient, who has developed a complication, and I need your help." That sentence conveys ownership, candor, and respect. The consultant can answer with an equally important sentence: "Tell me the timeline, what you have tried, what you are worried about, and what decision you need from me." Those two sentences can change the culture of a complication consult.

The patient-facing standard should be similarly direct: "This is a serious problem, and we are adding the right expertise." The chart standard should be similarly objective: "The patient has exposed hardware with surrounding nonviable tissue after prior fixation; orthopedic and plastic surgery discussed staged debridement, culture-directed antibiotics, and delayed coverage planning." None of these statements hides the complication, attacks the surgeon, or abandons the patient.

The five-part etiquette framework

A simple framework is useful. The first element is candor: name the complication accurately. The second is courtesy: communicate directly, early, and at the appropriate level. The third is continuity: the index surgeon remains involved even when another service assumes part of the care. The fourth is completeness: records, imaging, cultures, operative details, photographs when appropriate, and contingency plans accompany the consult. The fifth is co-ownership: the complication becomes a shared clinical problem, not the property of whichever service was called last.

This framework is intentionally simple because complication etiquette must work at night, in the operating room, on weekends, across institutions, and under stress. The more complex the case, the more important the etiquette becomes.

Conclusions

Plastic surgeons do not object to difficult consults. Difficult reconstruction is part of the specialty. What undermines care is late, vague, incomplete, or blame-shifting consultation that asks the consultant to solve a technical problem while the patient experiences abandonment. The complication consult should instead be treated as an escalation of care grounded in candor, direct communication, continued ownership, objective documentation, and shared responsibility.

Etiquette after a surgical complication is not superficial politeness. It is patient safety, professionalism, and trust made visible. When surgeons call early, speak honestly, preserve reconstructive options, remain involved, and protect the patient from interservice blame, they model the culture that patients deserve and that trainees should inherit.
